# Overexpression of RORγt Enhances Pulmonary Inflammation after Infection with *Mycobacterium Avium*

**DOI:** 10.1371/journal.pone.0147064

**Published:** 2016-01-19

**Authors:** Masashi Matsuyama, Yukio Ishii, Hirofumi Sakurai, Satoshi Ano, Yuko Morishima, Keigyou Yoh, Satoru Takahashi, Kenji Ogawa, Nobuyuki Hizawa

**Affiliations:** 1 Department of Respiratory Medicine, Division of Clinical Medicine, University of Tsukuba, Tsukuba, Japan; 2 Department of Nephrology, Division of Clinical Medicine, University of Tsukuba, Tsukuba, Japan; 3 Department of Anatomy and Embryology, University of Tsukuba, Tsukuba, Japan; 4 International Institute for Integrative Sleep Medicine, Life Science Center, University of Tsukuba, Tsukuba, Japan; 5 Tsukuba Advanced Research Alliance, University of Tsukuba, Tsukuba, Japan; 6 Laboratory Animal Resource Center, University of Tsukuba, Tsukuba, Japan; 7 Department of Clinical Research, National Hospital Organization, Higashinagoya National Hospital, Nagoya, Japan; Louisiana State University, UNITED STATES

## Abstract

*Mycobacterium avium* complex (MAC) is the most common cause of nontuberculous mycobacterial disease in humans. The role of Th17 immunity in the pathogenesis of intracellular bacteria, such as MAC, is not currently understood. Transcription factor RAR-related orphan receptor gamma t (RORγt) is known as the master regulator for Th17 cell development. Here, we investigated the role of RORγt in host responses against MAC infection. Wild-type (WT) mice and *RORγt*-overexpressing mice were infected with MAC via intratracheal inoculation. Systemic MAC growth was not different between WT mice and *RORγt*-overexpressing mice. However, neutrophilic pulmonary inflammation following MAC infection was enhanced in *RORγt*-overexpressing mice compared with that in WT mice. The cytokine expression shifted toward a Th17 phenotype in the lungs of *RORγt*-overexpressing mice following MAC infection; the levels of IL-6 and IL-17 were significantly higher in the lung of these mice than in WT mice. In addition to the increase in IL-17 single-positive T cells, T cells producing both IL-17 and interferon-γ were elevated in the lung of *RORγt*-overexpressing mice following MAC infection. These findings suggest that RORγt overexpression-mediated Th17 bias contributes to local inflammation rather than systemic responses, by regulating neutrophil recruitment into the sites of infection during MAC infection.

## Introduction

Nontuberculous mycobacteria (NTM) are an important cause of morbidity and mortality in pulmonary infectious diseases. The prevalence of NTM is increasing worldwide, especially in industrialized countries [1]. *Mycobacterium avium* complex (MAC) is the most common NTM that causes disease in humans [2]. Pulmonary MAC disease is divided into two forms: the primary form usually develops in nonsmoking post-menopausal women without known antecedent pulmonary disease, while the secondary form usually develops in patients with underlying pulmonary diseases, including old tuberculosis and bronchiectasis [3]. The factors predisposing to pulmonary MAC infection are not well understood, but some host factors may regulate susceptibility to pulmonary MAC disease.

T cell immunity is thought to be an important host factor regulates MAC susceptibility because disseminated MAC disease is often developed in patients with acquired immunodeficiency syndrome (HIV/AIDS). Among the T cells, type 1 T (Th1) cell-mediated immune responses play a central role in providing protection against intracellular pathogens, including MAC. This is because the Th1 cytokine interferon-gamma (IFN-γ) activates nitric oxide production in macrophages, which subsequently enhances mycobactericidal activities [4]. It is thought that susceptibility to mycobacteria could be explained by an immune dominance of either a Th1 or Th2 phenotype because Th2 cytokines interleukin (IL)-4 and IL-13 inhibit Th1-mediated mycobactericidal activity [5]. However, the majority of studies failed to reveal the presence of mycobacteria-specific Th2 cells [6,7].

The transcription factor T-box expressed in T cells (T-bet) is known as the critical regulator of Th1 differentiation and Th1 cytokine production [8]. We recently demonstrated that T-bet-overexpressing mice were resistant to pulmonary MAC infection and their cytokine expression was shifted toward Th1 phenotype [9]. However, T-bet-deficient mice were susceptible to MAC and had a higher expression of Th17 cytokines, such as IL-6 and IL-17. These findings suggest that the Th1/Th17 balance is a more critical determinant for host resistance to MAC infection than the Th1/Th2 balance. It is generally accepted that Th17 cells participate in host defense against fungi and extracellular bacteria [10]. As for their role in defending against intracellular pathogens, Ross and colleague have demonstrated that Th17 responses protectively contribute to host immunity against infection with *Bordetella pertussis* [11]. However, the role of Th17 immunity in host defense against mycobacteria is not fully understood. The transcription factor RAR-related orphan receptor gamma t (RORγt), a member of the nuclear receptor superfamily, was recently described as a master regulator for Th17 differentiation under the influence of several cytokines, such as transforming growth factor (TGF)-β, IL-6, IL-1β, and IL-23 in mice and humans [12–14]. We recently generated transgenic mouse overexpressing RORγt specifically in lymphocytes (*RORγt-tg* mouse) [15,16]. Using these mice, we investigated the role of RORγt in the susceptibility to MAC disease.

## Materials and Methods

### Ethics Statement

All animal procedures were performed in accordance with the University of Tsukuba guidelines for proper conduct of animal experiments. All animal studies were approved by the Institutional Review Board of the University of Tsukuba (permit number: 13–093).

### Mycobacteria

A clinically isolated *M*. *avium subsp*. *hominissuis* strain obtained from a non-HIV-infected patient (TH48) was used in this study. The mycobacteria were grown to mid-log phase in Middlebrook 7H9 liquid medium (Difco/Becton Dickinson), aliquoted, and frozen at -80°C until use. Bacterial counts in each organ were determined by plating serial dilutions of organ homogenates from individual mice onto Middlebrook 7H10 agar plates and counting the number of bacterial colonies present two weeks after the infection. The number of colony-forming units (CFU) is expressed as the mean CFU from eight individual mice.

### Mice and Infection

Wild-type (WT) C57BL/6 mice were purchased from Charles River (Yokohama, Japan). *RORγt-tg* mise under the control of the CD2 promoter were generated as previously described, and we confirmed that transgenic *RORγt-tg* mRNA is expressed in the T cells but not in the B cells or CD11b-positive macrophages in these mice [15]. Female mice (8 to 12 weeks-old) were used in all experiments. Mice were anesthetized with isoflurane and intubated orotracheally with a 22-gauge intravenous catheter, followed by the administration of 1x10^7^ CFU of *M*. *avium* in 50 μl sterile saline. Control mice were treated with 50 μl saline.

### Histology

Mice were euthanized 2 months after MAC infection. Lungs were removed and fixed with 10% neutral buffered formalin at 25 cm H_2_O pressure for 48 hours, washed with phosphate-buffered saline (PBS), processed, and embedded in paraffin. The paraffin blocks were sectioned into 2-μm sections for histopathological analysis. The sections were then deparaffinized, hydrated, and stained with hematoxylin and eosin (H&E) as well as with a Ziehl-Neelsen reagent to identify the bacilli. Inflammation in lung sections was semi-quantitatively graded for severity by scanning multiple random fields in three sections of lung tissue per mouse: 0 = no lesion, 1 = minimal lesion(s) (1–10% of the involved area), 2 = mild lesion(s) (11–30% of the involved area), 3 = moderate lesion(s) (31–50% of the involved area), 4 = marked lesion(s) (50–80% of the involved area), 5 = severe lesion(s) (>80% of the involved area) [17].

### Bronchoalveolar Lavage (BAL)

The trachea of deeply anesthetized mice by pentobarbital was exposed and an 18 gauge teflon tube was inserted into the trachea. The lungs were then lavaged with six sequential 1-ml aliquots of saline. BAL fluids were collected into 15-ml conical centrifuge tube (Thermo Scientific) and centrifuged at 1500 rpm for 5 minutes. The cell pellets were resuspended with PBS containing 0.1 mM EDTA. The cells were then counted using a hemocytometer, and differential cell counts were obtained after staining with Diff-Quick (Polysciences, Inc.).

### Quantitative reverse transcription-Polymerase Chain Reaction (qRT-PCR)

Total RNA was extracted from lungs tissues using RNeasy Mini Kit (Qiagen Inc) according to the manufacturer's instructions. qRT-PCR was performed using a sequence detector (ABI7700; Applied Biosystems) according to the manufacturer's instructions. The PCR primers used in this study are listed in Table 1. The gene expression levels for each amplicon were calculated using the ∆∆CT method and normalized against glyceraldehydes 3-phosphate dehydrogenase (GAPDH) mRNA expression.

**Table 1:** Primers used for RT-PCR.

Primer target	Sequence
GAPDH	5’-CCGCATCTTCTTGTGCAGTG-3’ (forward),
	5’-CGTTGATGGCAACAATCTCC-3’ (reverse)
IFN-γ	5’-CACGGCACAGTCATTGAAAG-3’ (forward),
	5’-TCTGGCTCTGCAGGATTTTC-3’ (reverse)
IL-17	5’-AAAGCTCAGCGTGTCCAAAC-3’ (forward),
	5’-TGGAACGGTTGAGGTAGTCTG-3’ (reverse)
IL-4	5’- ACGGAGATGGATGTGCCAAAC-3’ (forward),
	5’- AGCACCTTGGAAGCCCTACAGA-3’ (reverse)
IL-6	5’-TAGTCCTTCCTACCCCAATTTCC-3’ (forward),
	5’-TTGGTCCTTAGCCACTCCTTC-3’ (reverse)
IL-10	5’- GCTCTTACTGACTGGCATGAG-3’ (forward),
	5’- CGCAGCTCTAGGAGCATGTG-3’ (reverse)
IL-23p19	5’- ATGCTGGATTGCAGAGCAGTA -3’ (forward),
	5’- ACGGGGCACATTATTTTTAGTCT -3’ (reverse)
IL-12p40	5’- TGGTTTGCCATCGTTTTGCTG -3’ (forward),
	5’- ACAGGTGAGGTTCACTGTTTCT -3’ (reverse)
TNF-α	5’-CCCTCACACTCAGATCATCTTCT-3’ (forward),
	5’-GCTACGACGTGGGCTACAG-3’ (reverse)

### Fluorescence-Activated Cell Sorting (FACS)

The lungs were removed 2 months after infection and digested with 75 U/ml collagenase (type 1; Sigma) at 37°C for 90 minutes. Isolated cells were filtered through a 20-μm nylon mesh and then stained with anti-CD4, anti-CD8, anti-CD3 and anti-TCRβ antibodies (Biolegend) to detect T cell subsets and analyzed by flow cytometry. T cell cytokine production was determined by flow cytometric intracellular cytokine analysis as described previously [18]. Briefly, cells were suspended at 10^6^/ml in RPMI 1640 containing 10% fetal calf serum, incubated with phosphomolybdic acid (50 ng/ml; Sigma) and ionomycin (500 ng/ml; Sigma) for 2 hours, and then incubated with brefeldin A (10 μg/ml; Sigma) for 2 hours at 37°C. Next, the cells were washed in PBS and fixed with 2% formaldehyde in PBS for 15 minutes at room temperature. The fixed cells were washed in PBS supplemented with 0.5% bovine serum albumin (BSA) and 0.02% sodium azide (PBS/BSA/azide). For intracellular cytokine detection, the cells were permeabilized with 0.5% saponin (Sigma) in PBS/BSA/azide, stained with PE-conjugated anti-mouse IFN-γ (Biolegend), PE-conjugated anti-mouse T-bet (eBioscience), PE-conjugated or APC-conjugated anti-mouse IL-17A (BD PharMingen), or APC-conjugated anti-mouse RORγT (eBioscience).

### Statistical Analysis

Data are expressed as the mean ± SEM. Data comparisons among the experimental groups were performed using a one-way ANOVA followed by post-hoc tests. Survival data were analyzed by a Kaplan-Meier and log-rank test. Values of *p* < 0.05 were considered to be statistically significant.

## Results

### RORγt Overexpression Exhibits Limited Effects on Systemic MAC Infection

**Fig 1:**
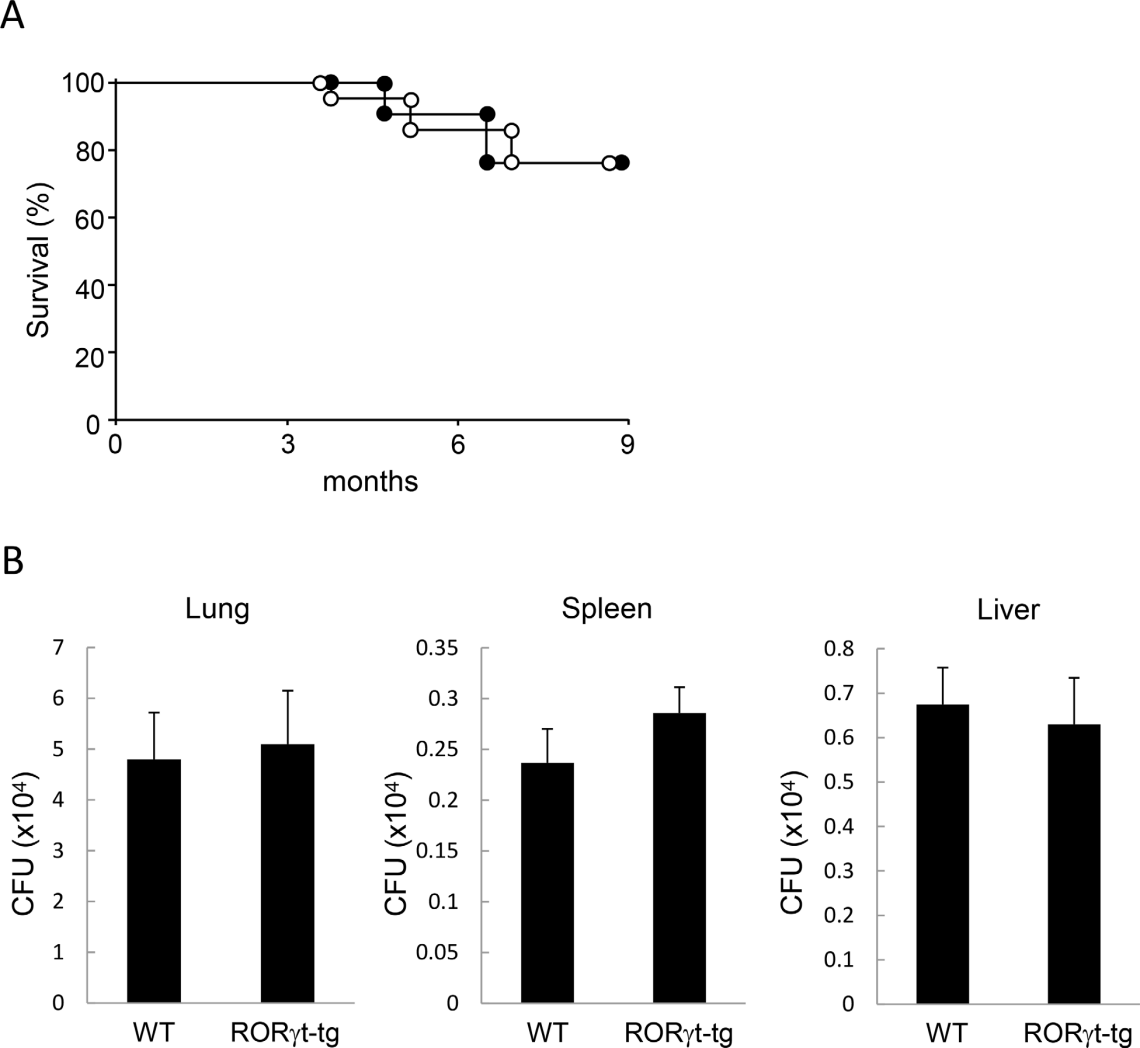
Responses to MAC in WT and RORγt-tg mice. (**A**) Survival of WT mice (filled circles) and *RORγt-tg* mice (open circles) after intratracheal inoculation of 1 x 10^7^ CFU of MAC or saline. n = 20 in each group. (**B**) Mycobacteria outgrowth in the lungs, spleens, and livers of WT and *RORγt-tg* mice 2 months after intratracheal inoculation of 1 x 10^7^ CFU of MAC. The results are expressed as CFU per organ. The experiments were performed in duplicate with eight mice in each group. Data are expressed as the mean ± SEM.

To assess the influence of RORγt on MAC susceptibility, we first evaluated the survival of WT mice and *RORγt-tg* mice 9 months after MAC infection. More than 70% of mice in both groups survived throughout the observation period. Notably, the survival rate was not different between WT mice and the *RORγt-tg* mice (Fig 1A).

We next evaluated the mycobacterial burden in WT mice and *RORγt-tg* mice following MAC infection. Intratracheal administration of 1x10^7^ CFU of *M*. *avium* caused systemic infection in both genotypes. Two months after MAC infection, mycobacterial growth was detected in the lungs, spleens, and livers of both genotypes. Measurement of organ CFU revealed that mycobacterial counts in these tissues were not different between WT mice and *RORγt-tg* mice (Fig 1B). These results indicate that RORγt-mediated host responses do not influence the growth in organs or its systemic spread.

### RORγt Overexpression Enhanced MAC-Induced Pulmonary Inflammation

**Fig 2:**
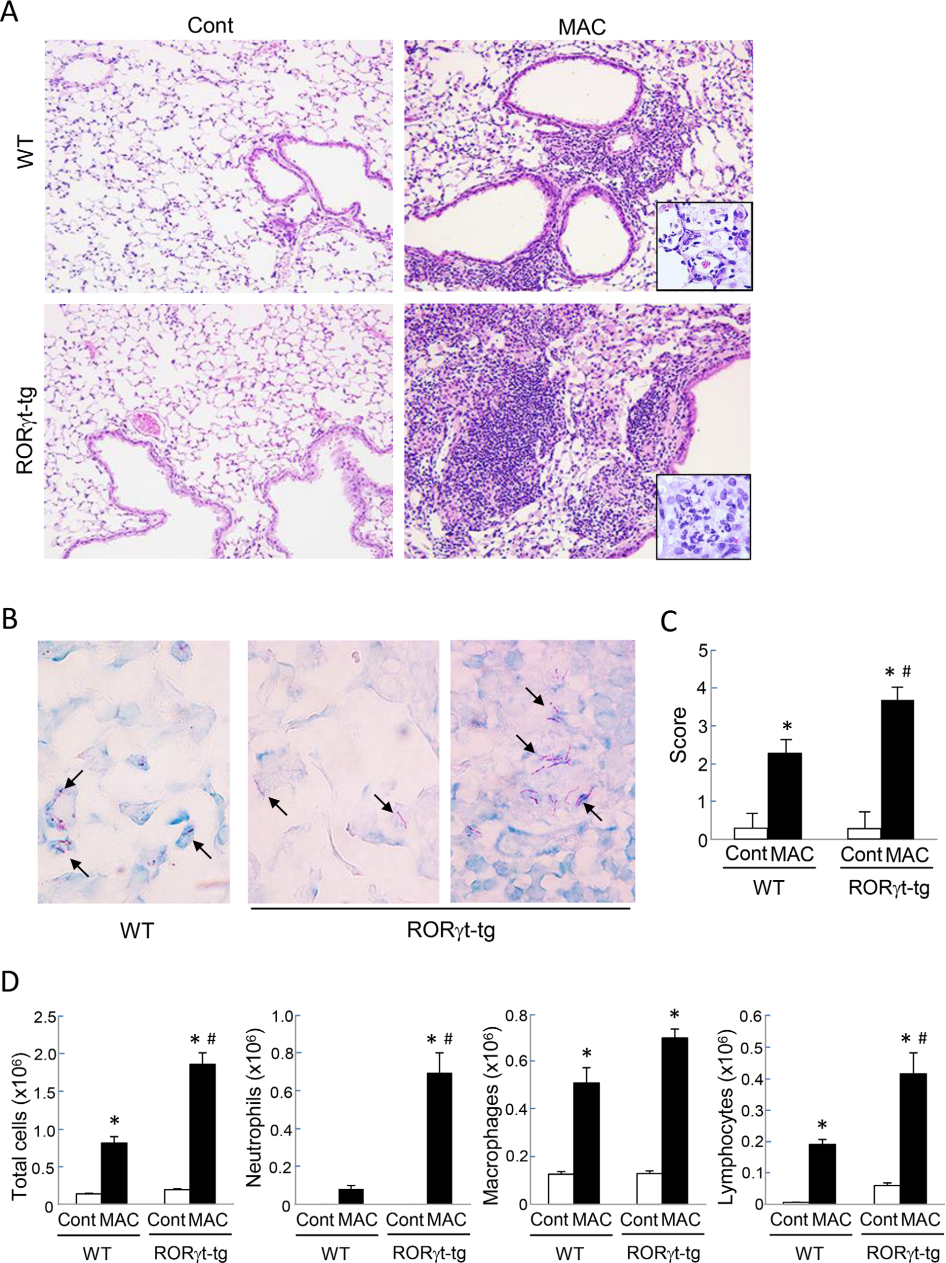
MAC-induced pulmonary inflammation in WT and RORγt-tg mice. (**A**) Representative microphotographs of lungs from WT and *RORγt-tg* mice 2 months after the intratracheal inoculation of 1 x 10^7^ CFU of MAC or saline (Cont). Magnification, x100. *Insets* show the inflammatory lesions at higher magnifications. (**B**) Representative photographs of Ziehl-Neelsen staining of an alveolar region from WT mice (left panel), and alveolar (center panel) and peribronchial (right panel) regions of *RORγt-tg* mice 2 months after the intratracheal inoculation of 1 x 10^7^ CFU of MAC. Arrows indicate acid-fast bacilli. Magnification, x400. (**C**) Semi-quantitative scoring of inflammation in the lungs of WT and *RORγt-tg* mice 2 months after intratracheal inoculation of 1 x 10^7^ CFU of MAC or saline (Cont). (**D**) The number of total cells, neutrophils, macrophages, and lymphocytes in BAL fluids from WT and *RORγt-tg* mice 2 months after the intratracheal inoculation of 1 x 10^7^ CFU of MAC or saline (Cont). All experiments were performed in duplicate with four mice in each group. *Significant difference between MAC and Cont group (p<0.05). #Significant difference between genotypes after MAC infection (p<0.05). Data are expressed as the mean ± SEM.

We histopathologically evaluated the MAC-induced pulmonary inflammation in both WT mice and *RORγt-tg* mice. Inflammatory cell infiltration was observed in peribronchial regions of WT mice 2 months after MAC infection (Fig 2A). In *RORγt-tg* mice, the inflammatory cell infiltration, especially the neutrophil infiltration, was more severe than in the WT mice, and it extended to the perivascular and alveolar regions (Fig 2A). No abnormal findings were observed in saline-administrated controls (Fig 2A). We then evaluated the distribution of mycobacteria in the lung tissues of both WT mice and *RORγt-tg* mice. Two months after MAC infection, acid-fast bacilli were most prominently observed in macrophages in the alveolar region of both genotypes (Fig 2B). The bacilli were also detected in granuloma-like lesions, where macrophages accumulated, located in peribronchial inflamed sites of *RORγt-tg* mice (Fig 2B). The lung inflammation was semi-quantitatively evaluated in both genotypes by using a scoring method. After MAC infection, the lung histological inflammation score was significantly higher in *RORγt-tg* mice than in WT mice (Fig 2C). Further, we quantitatively evaluated the degree of pulmonary inflammation in this infection model with both WT mice and *RORγt-tg* mice by assessing the number of cells recovered from the BAL. The number of BAL-recovered inflammatory cells was increased following MAC infection in both genotypes. Additionally, the numbers of neutrophils and lymphocytes were significantly higher in the BAL fluids of *RORγt-tg* mice than those in the BAL fluids of WT mice (Fig 2D). These results indicate that RORγt overexpression enhances MAC-induced pulmonary inflammation, particularly neutrophilic inflammation.

### RORγt Overexpression Induces Th17 Cytokines in Lung Tissues after MAC Infection

**Fig 3:**
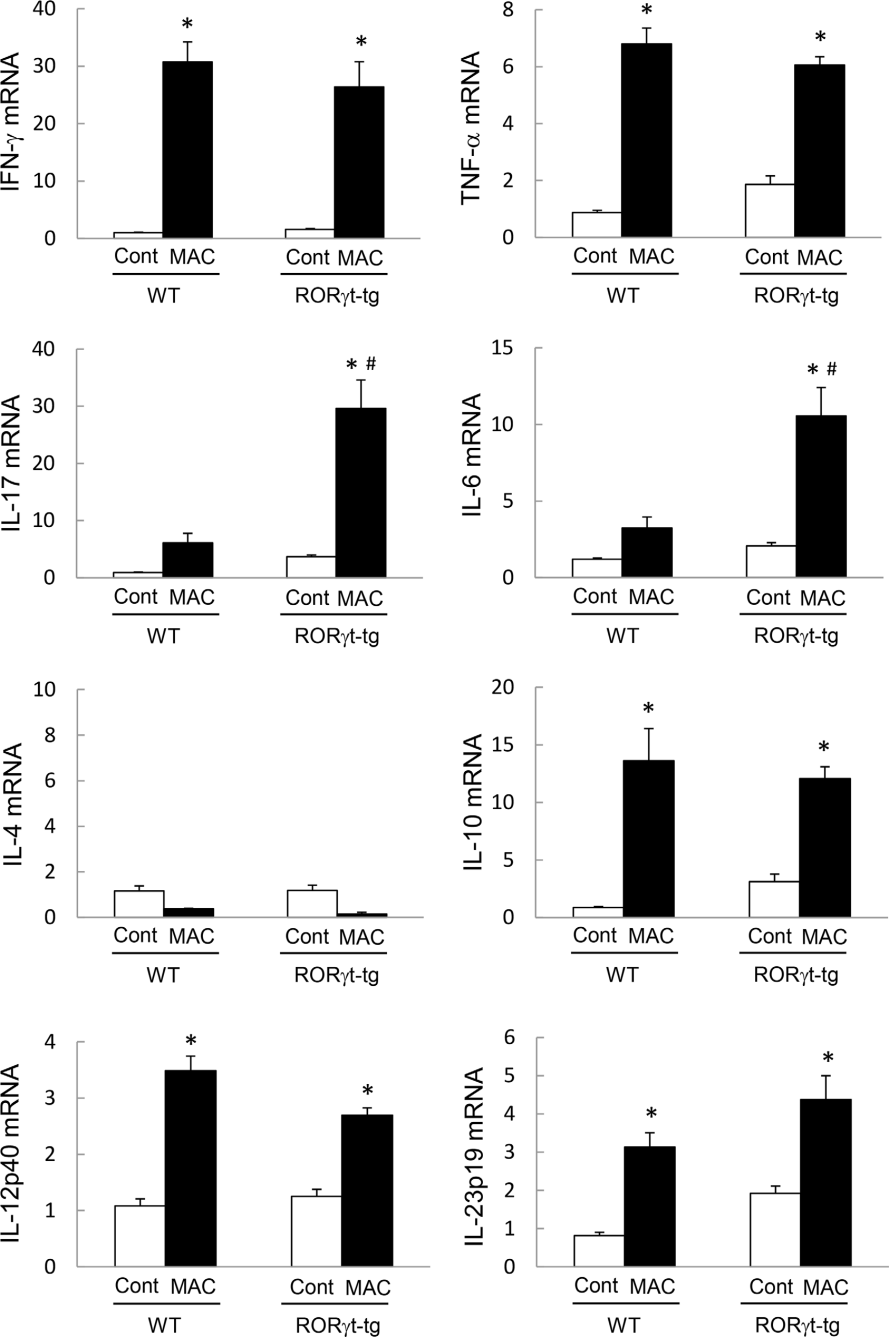
Pulmonary cytokine expression in WT and RORγt-tg mice after MAC Infection. The expression of IFN-γ, TNF-α, IL-4, IL-6, IL-10, IL-12p40, IL-17, and IL-23p19 in the lungs of WT and *RORγt-tg* mice 2 months after the intratracheal inoculation of 1 x 10^7^ CFU of MAC or saline (Cont). The expression of each mRNA was analyzed by qRT-PCR, and the y-axis of each graph represents the relative expression of the respective genes calculated using the ∆∆CT method and normalized against GAPDH mRNA. Experiments were performed in duplicate with five mice in each group. *Significant difference between MAC and Cont group (p<0.05). #Significant difference between genotypes after MAC infection (p<0.05). Data are expressed as the mean ± SEM.

Because RORγt is known as the critical regulator of Th17 cell differentiation and Th17 cytokine expression, we next assessed the cytokine expression in the lungs of WT mice and *RORγt-tg* mice 2 months post-MAC infection. Although the expression of IFN-γ and tumor necrosis factor-α (TNF-α) increased after MAC infection in the lungs of both WT mice and *RORγt-tg* mice, the expression level was not different between the genotypes (Fig 3). The levels of lung IL-17 and IL-6 expression also increased in both genotypes after MAC infection. However, in MAC-infected mice, the levels of IL-17 and IL-6 expression were significantly higher in *RORγt-tg* mice than in WT mice (Fig 3). IL-4 expression was not induced in the lungs of any mice following MAC infection (Fig 3). IL-10, IL-12, and IL-23 expressions were induced significantly in the lungs of WT mice and *RORγt-tg* mice after MAC infection (Fig 3), but the expression levels of these cytokines were not different between the genotypes. These results indicate that Th1 cytokines are induced in the lungs of both WT mice and *RORγt-tg* mice after MAC infection. In contrast, Th17 cytokines are specifically and strongly induced in the lung of *RORγt-tg* mice.

### RORγt Overexpression Enhances IL-17 Production in Lung T Cells

**Fig 4:**
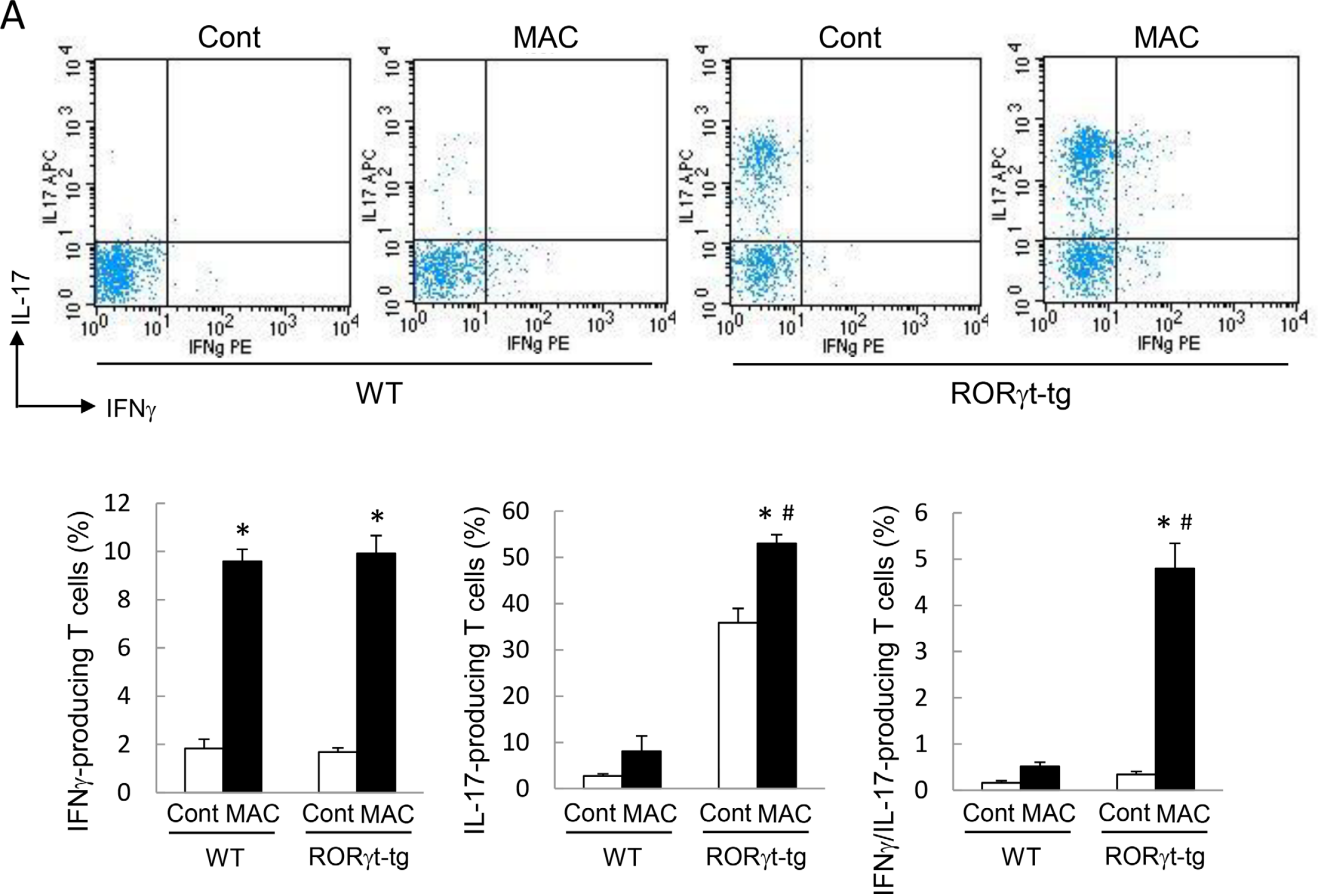
IL-17-producing T cells in the lungs of WT and RORγt-tg mice after MAC infection. The proportion of IL-17- and IFN-γ-producing cells in CD4-positive T cells obtained from the lungs of WT and *RORγt-tg* mice 2 months after intratracheal inoculation of 1 x 10^7^ CFU of MAC or saline (Cont). The IFN-γ- and IL-17-positive cells were detected by FACS using PE-conjugated anti-mouse IFN-γ and APC-conjugated anti-mouse IL-17 antibodies. Representative plots (upper panel) and mean value among triplicate samples (lower panel) are shown. *Significant difference between MAC and Cont group (p<0.05). #Significant difference between genotypes after MAC infection (p<0.05). Data are expressed as the mean ± SEM.

We assessed the production of IFN-γ and IL-17 in CD4-positive T cells obtained from the lungs of WT mice and *RORγt-tg* mice to clarify the contribution of CD4-positive T cells to Th1 and Th17 cytokine production. The number of IFN-γ-producing CD4-positive T cells increased in the lungs of both genotypes after MAC infection (Fig 4). In the MAC-infected mice, the amount of IFN-γ-producing CD4 positive T cells was not different between the genotypes. The proportion of IL-17-producing CD4-positive T cells was significantly higher in the lung of *RORγt-tg* mice than in the lungs of WT mice, regardless of MAC infection (Fig 4). In *RORγt-tg* mice, the proportion of IL-17-producing cells was significantly elevated after MAC infection, compared with that in uninfected *RORγt-tg* mice (Fig 4). The proportion of IFN-γ/IL-17-coproducing CD4-positive T cells increased significantly in the lungs of *RORγt-tg* mice after MAC infection, compared with that in the lungs of uninfected *RORγt-tg* mice (Fig 4).

**Fig 5:**
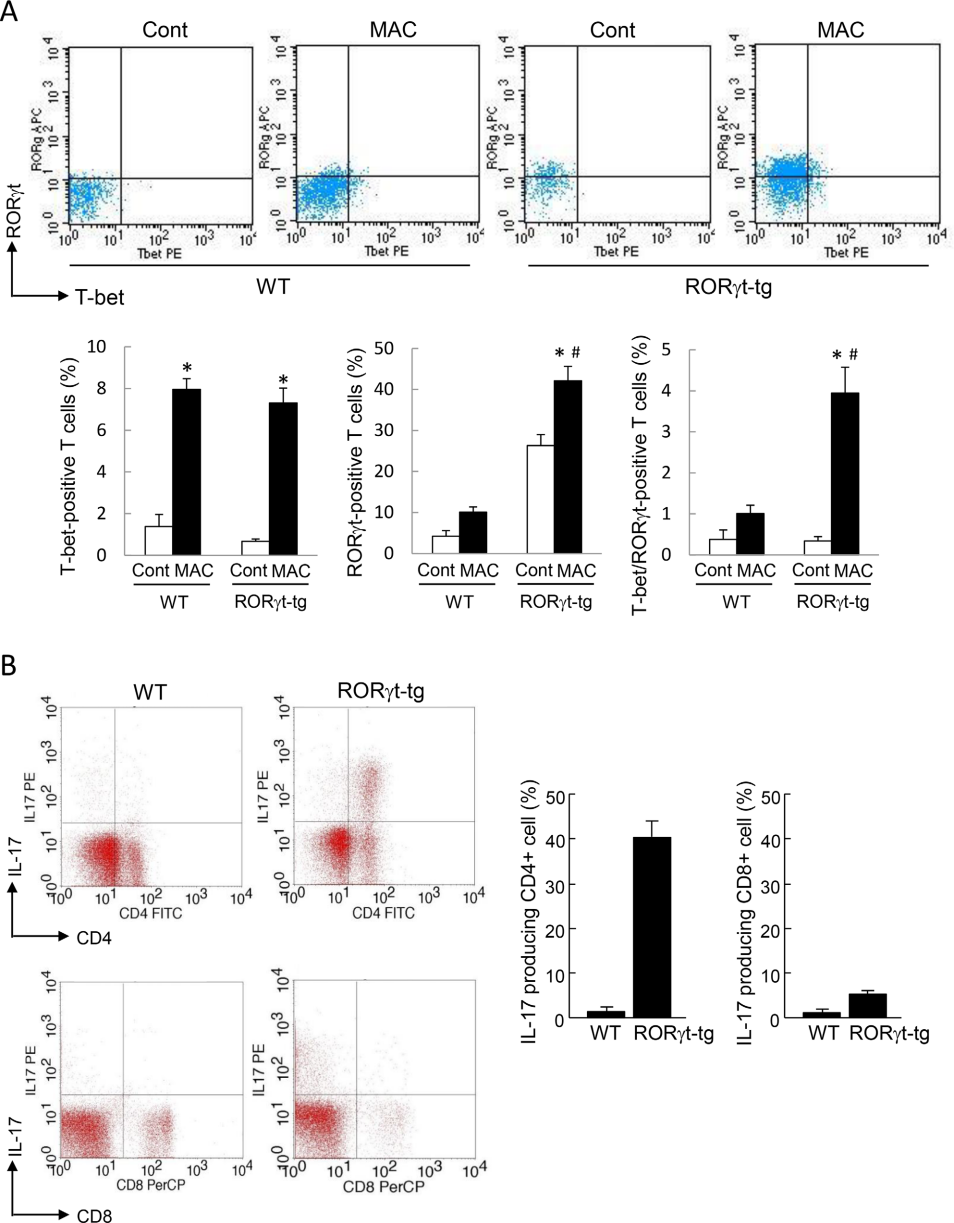
RORγt-expressing T cells in the lungs of WT and RORγt-tg mice after MAC infection. **(A)** The proportion of CD4-positive T cells expressing RORγt and/or T-bet in the lungs of WT and *RORγt-tg* mice 2 months after an intratracheal inoculation of 1 x 10^7^ CFU of MAC or saline (Cont). The T-bet- and RORγt-positive cells were detected by FACS using PE-conjugated anti-T-bet and APC-conjugated anti-RORγt antibodies. Representative plots (upper panel) and mean value among triplicate samples (lower panel) are shown. **(B)** The proportion of IL-17-producing cells in CD4-positive (upper panels) and CD8-positive T cells (lower panels) obtained from the lungs of WT and *RORγt-tg* mice 2 months after an intratracheal inoculation of 1 x 10^7^ CFU of MAC. The IL-17-, CD4-, and CD8-positive cells were detected by FACS using PE-conjugated anti-IL-17, FITC-conjugated anti-CD4, and PerCP-conjugated anti-CD8 antibodies. Representative plots (left panel) and mean value among duplicate samples (lower panel) are shown. *Significant difference between the MAC and Cont group (p<0.05). #Significant difference between genotypes after MAC infection (p<0.05). Data are expressed as the mean ± SEM.

To clarify the distribution of the T cell subsets, we then assessed the proportion of T-bet-positive T cells and RORγt-positive T cells in the lungs of WT mice and *RORγt-tg* mice. Similar to the cytokine production results, the number of T-bet-positive T cells increased in the lungs of both genotypes after MAC infection (Fig 5A). In the MAC-infected mice, the proportion of T-bet-positive lung T cells was not different between the genotypes. The proportion of RORγt-positive lung T cells was significantly higher in *RORγt-tg* mice than in WT mice, regardless of MAC infection (Fig 5A). In *RORγt-tg* mice, the proportion of RORγt-positive lung T cells was significantly elevated after MAC infection, relative to the proportion in uninfected *RORγt-tg* mice (Fig 5A). The proportion of T-bet/RORγt-double positive lung T cells increased significantly in the lung of *RORγt-tg* mice after MAC infection, compared with the proportion in uninfected *RORγt-tg* mice (Fig 5A). These results indicate that the balance of the immune response in the lung is shifted toward a Th17 phenotype in *RORγt-tg* mice after MAC infection.

To clarify the contribution of CD8-positive T cells to the Th17 bias that we observed in this infection model, the IL-17 production was evaluated in the CD4-positive T cells and CD8-positive T cells obtained from the lungs of WT mice and *RORγt-tg* mice 2 months following MAC infection. As stated above, IL-17-producing CD4-positive cells increased markedly after MAC infection (Fig 5B). However, a low level of IL-17-producing CD8-positive cells was observed, and there was no significant difference in the level of these cells between the WT mice and *RORγt-tg* mice after MAC infection (Fig 5B). These results indicate that CD8-positive T cells are not strongly involved in the generation of a Th17 bias in MAC-infected *RORγt-tg* mice.

## Discussion

In response to antigen, such as those from microorganisms, naïve CD4-positive T cells can be differentiated into different T cell subpopulations, including Th1, Th2, Th17, and regulatory T (Treg) cells depending on the cytokine milieu to which they are exposed. RORγt is a transcription factor belonging to a large family of hormone nuclear receptors and is known as a lineage-specific transcription factor for the development of Th17 cells [12–14,19,20]. RORγt is expressed in Th17 cells and directs the transcriptional activation of the *IL-17* gene, which is responsible for the lineage-specific cytokine of the Th17 cells [14,21,22]. In the present study, higher levels IL-17 expression were observed in the lung CD4-positive T cells of *RORγt-tg* mice than in those of WT control mice, under both uninfected and MAC-infected conditions. We also demonstrated that the amount of RORγt-expressing T cells was higher in the lungs of *RORγt-tg* mice than in the lungs of WT mice. These findings suggest that the lung Th balance is shifted toward Th17 phenotype in our transgenic mice that overexpress RORγt. It is unclear why all the T cells in *RORγt-tg* mice do not express RORγt because the expression of the RORγt transgene is under the control of the CD2 promoter. Potential explanations for our findings are the detection limit of FACS or an inactivation of RORγt through interaction with other lineage-specific transcription factors during T cell development.

It is generally accepted that Th17 immunity plays a central role in the protection against fungi and extracellular bacteria [10,23]. However, the role of Th17 in regulating intracellular pathogens, such as mycobacteria, is not fully understood. Initial studies suggested that the IL-17/Th17 pathway was not essential for protection against mycobacteria, such as *M*. *tuberculosis* and *M*. *bovis* [24]. However, recent studies demonstrated that the IL-17/Th17 pathway may play a role in anti-mycobacterial immunity by recruiting neutrophils to the site of infection at the early stage of tuberculosis [25,26], or by accelerating the accumulation of Th1 cells and enhancing Th1 anti-mycobacterial responses [26–28]. IL-17 also plays a role in the formation and maintenance of granulomas in mycobacteria-infected lungs [29,30]. Re-exposure of tuberculosis-infected mice to high levels of tuberculosis antigen promotes further Th17 responses that cause extensive lung damage, which is associated with elevated neutrophil recruitment [31]. We demonstrated that the numbers of mycobacteria in several organs were not different in *RORγt-tg* mice compared with the corresponding values in WT control mice, even though the concentration of IL-17 was significantly elevated in response to MAC infection. Correspondingly, we previously demonstrated that IL-17 neutralization did not exacerbate the bacterial burden in Th17-biased T-bet-deficient mice [9]. Taken together, it is likely that an increase in IL-17/Th17 responses does not directly link to the enhancement of anti-mycobacterial activity in our MAC infection model.

In the present study, we demonstrated that neutrophilic pulmonary inflammation was enhanced in the lungs of *RORγt-tg* mice following MAC infection. IL-17 is considered to be an important mediator for neutrophilic inflammation that acts by inducing the production of GM-CSF, which activates neutrophil differentiation [32], and the production of neutrophil attractant CXC chemokines [22,33,34]. Therefore, an enhancement of neutrophilic pulmonary inflammation may be associated with an increase in the IL-17 level in *RORγt-tg* mice following MAC infection. The findings in our previous study, that neutralization of IL-17 clearly attenuated MAC-induced neutrophil recruitment in Th17-biased T-bet-deficient mice, support the hypothesis [9]. In patients without immunodeficiencies, neutrophils were the main cellular constituents in BAL fluids during pulmonary MAC infection [35]. Neutrophilic pulmonary inflammation with decreased CD4-positive lymphocytes reflected disease progression in these patients [36]. In our MAC infection model, neutrophils were not essential for mycobacterium killing because organ MAC CFUs were not different between *RORγt-tg* and WT mice. Thus, enhanced neutrophil recruitment derived from Th17 deviation might have pathological, rather than protective, effects during MAC infection in our model.

It is generally accepted that lineage-specific transcription factors can inhibit the differentiation of other Th subsets. In fact, we previously demonstrated that T-bet suppresses IL-17 production and Th17 cell differentiation by controlling the nitric oxide level after MAC infection [9]. However, in the present study, lung IFN-γ level was not suppressed in *RORγt-tg* mice following MAC infection. We also found that CD4-positive T cells producing both IL-17 and IFN-γ increased in *RORγt-tg* mice following MAC infection. Previous studies have demonstrated that, in addition to cells producing either IL-17 or IFN-γ, elevated numbers of IL-17/IFN-γ double-positive T cells were observed in both human and mouse inflamed tissues [37,38]. It was also reported that CD4-positive cells that express both IFN-γ and IL-17 were observed in the peripheral blood and pleural fluid from patients with tuberculosis [39]. This suggests a complex process of transcription factor-regulated T cell differentiation during infection. Boniface *et al*. have demonstrated that the development of IL-17/IFN-γ double-positive T cells is under the influence of RORγt and that these cells may belong to the Th17 lineage but they are distinct from Th1 lineage [40]. The expression analysis of the transcription factor revealed that, equal with the increase in IL-17/IFN-γ double-positive T cells, T cells co-expressing RORγt and T-bet increased following MAC infection.

It has been reported that Th17 cells may represent a heterogeneous population with distinct trafficking profiles and differing abilities. Ghoreschi *et al*. have demonstrated that Th17 cells express both RORγt and T-bet when these cells are generated in the absence of TGF-β [41]. RORγt and T-bet double-positive Th17 cells were present *in vivo* in lesional tissue in experimental allergic encephalomyelitis [41] and multiple sclerosis [38]. These cells might be more relevant to the pathogenesis of diseases that develop both Th1- and Th17-mediated pathology. The role of RORγt and T-bet double-positive T cells in the pathogenesis of MAC disease should be elucidated in the future.

In summary, lymphocyte-restricted overexpression of RORγt induced a Th17 bias in the lung tissue following MAC infection. This RORγt-mediated Th17 bias did not affect the systemic growth of MAC, whereas it enhanced the neutrophilic pulmonary inflammation following MAC infection. Excessive Th17 responses might produce pathological effects, rather than provide protection, during MAC infection. The lung histopathology in *RORγt-tg* mice resembled the histopathology in patients with pulmonary MAC disease. Therefore, we will examine the appearance of Th17 cells and the IL-17 level in the BAL fluids and lung tissues of patients with pulmonary MAC disease in our next study.
